# Metastases to the thyroid gland: ultrasonographic findings and diagnostic value of fine-needle aspiration cytology

**DOI:** 10.3389/fonc.2022.939965

**Published:** 2022-08-03

**Authors:** Zhenyun Tang, Lili Gao, Xue Wang, Jingwen Zhang, Weiwei Zhan, Wei Zhou

**Affiliations:** ^1^ Department of Ultrasound Diagnosis, Ruijin Hospital Affiliated to Shanghai Jiaotong University, Shanghai, China; ^2^ Department of Pathology, Ruijin Hospital Affiliated to Shanghai Jiaotong University, Shanghai, China

**Keywords:** secondary thyroid neoplasm, thyroid, metastasis to the thyroid gland, thyroid metastasis, ultrasound, fine-needle aspiration cytology

## Abstract

**Introduction:**

In the present study, we aimed to analyze ultrasonographic findings of metastases to the thyroid and explore the role of fine-needle aspiration cytology (FNAC) in the diagnosis of metastases to the thyroid.

**Methods:**

Twelve cases of cytologically or/and pathologically confirmed metastatic tumors of the thyroid gland were reviewed. All the primary thyroid lesions and lymphomas were excluded. The location, maximum size, echogenicity, shape, margin, presence of calcifications, vascularity, and cervical lymph nodes were assessed on ultrasonography. In addition, the results of cytology or pathology (or both) were noted retrospectively.

**Results:**

Eight of 10 patients were diagnosed correctly with FNAC. Two cases presented with diffuse involvement in both thyroid lobes. Nine cases demonstrated a hypoechoic nodule with an irregular margin, four of which had microcalcifications. One case presented with a mixed solid and cystic mass with an oval shape. The lesions with cervical lymph nodes were found in seven cases.

**Conclusion:**

Most metastatic thyroid cancer has similar ultrasound features to primary thyroid cancer. In some cases with atypical US features, ultrasound diagnosis should be combined with the medical history. FNAC might be helpful in the diagnosis.

## Introduction

Metastatic tumor to the thyroid gland (MTTG) is scarce (1). The sources of MTTG are various (2), including kidney, lung, breast, and gastrointestinal, which are the most common in the literature (3). The incidence of MTTG is 1.25-24% in autopsy series, and it is less common in clinical findings (4–6). It has been reported that MTTG accounted for 1.4-3% of all the patients who underwent surgery for thyroid malignancy (7). The difference in incidence between autopsy and clinical findings is partly attributed to the comparatively short survival of patients with advanced primary malignant tumors, which is not conducive to detecting newly detected thyroid masses (8).

The diagnosis of MTTG in patients with widespread metastases can avoid unnecessary operations. However, surgery is beneficial and may prolong life expectancy when the thyroid gland is isolated metastasis of non-thyroid malignancies during follow-up of indolent primary disease (7). Hence, it is essential to detect MTTG for patients with solitary thyroid metastasis who may benefit from thyroidectomy.

However, it is challenging to identify MTTG at the early stage due to the lack of specific symptoms. The concomitance of thyroid goiter can also cause a diagnostic dilemma (9). Therefore, MTTG may not be detected until a long time after the primary tumor is diagnosed (2). High-frequency ultrasound (US) is an essential tool to detect thyroid nodules, and it is also helpful in guiding fine-needle aspiration cytology (FNAC) for suspicious lesions (10). As an accurate and reliable technique, FNAC is considered the gold standard in preoperative procedures for various thyroid tumors (11). Several sporadic studies have analyzed clinical characteristics of MTTG (4, 9, 12, 13), while only a few studies have focused on the ultrasonographic features (8, 14, 15) or FNAC results (2, 16, 17) (or both (10, 11)). To expand these findings, we aimed to analyze the clinical data, ultrasonographic features, and the role of US-guided FNAC in detecting MTTG. These findings might help us further understand the US characteristics and accurately diagnose MTTG.

## Materials and methods

The study was approved by the ethics committee of our hospital, along with the waiver of consent to participate. The medical databases were retrospectively reviewed in a single territory hospital between January 2014 and December 2021. The patients enrolled in this study met the following criteria: (1) MTTG was diagnosed by cytological or histological pathology; (2) the patients had malignancies in other organs, and the primary malignancies were confirmed histologically; (3) all the patients underwent US examinations. The excluded criteria were set as follows: (1) metastases from lymphomas; (2) absence of US images. A total of 12 patients were finally included in our present study. The clinical data including the demographic characteristics and medical history were collected, which were obtained from the medical records.

US examination was performed with a 12-5 MHz linear array transducer (iU22; Philips Medical Systems) or a 14-5 MHz transducer (Resona 7, Mindray medical system, Shenzhen, China). The patients were examined in a supine position with hyperextension of the neck. Grayscale and color-Doppler ultrasonography were applied to evaluate the thyroid lesions.

The US features of thyroid lesions were evaluated as follows: forms (nodule or diffuse); locations (right, left, or isthmus of the thyroid); the maximum diameter of the lesion was measured if it was displayed as a nodule; composition (solid, mixed solid and cystic, or cystic); echoic pattern (hypo-, hyper-, or isoechoic compared with the surrounding thyroid parenchyma); margin (circumscribed or irregular); shape (parallel defined as wider than taller shape, or non-parallel defined as taller than wide shape); and presence of calcification (non, microcalcification, macrocalcification). Microcalcification was described as hyperechoic spots <1 mm with or without shadowing, vascularization on color Doppler (no flow signal, intra-nodular signal, or peripheral signal), degree of vascularization (none, low, middle, or high), presence of tumor thrombus in the internal jugular vein, presence of suspicious lymph nodes including at least one of the following criteria: round shape, focal or diffuse hyper-echogenicity, absence of fatty hilum, cystic component, and presence of internal calcification or chaotic vascularity. One radiologist with eight years of experience reviewed the US features.

FNAC was performed using a 25-27 gauge needle, and local anesthesia was not applied. At least two smears were aspirated for each thyroid lesion. The remaining samples in the syringe were dedicated to the cell block preparation for further cytological evaluations. Immunocytochemical analysis was performed by a group of commercially available antibodies if necessary. Core needle biopsy (CNB) was used in three patients with local anesthesia. An 18-gauge semi-automated core biopsy needle was applied in CNB. At least two passes were acquired for each thyroid nodule. Five patients finally underwent total thyroidectomies. The histological specimens were obtained from formalin-fixed, paraffin-embedded tissues before they were stained with hematoxylin and eosin (H&E) stain, followed by histological and immunocytochemical analyses.

## Results

The study consisted of eight females and four males, with a mean age of 60 (range, 48-69 years). Seven cases were incidentally detected during US or positron emission tomography-computed tomography (PET-CT) whole-body examinations, and 5 cases were found with symptoms. Of the five patients, one had hoarseness, and progressive enlargement of neck mass was observed in 4 cases, 2 of which had cough symptoms.

Of the 12 metastatic lesions, there were four breast malignancies, two renal cell carcinomas (RCC), two laryngeal carcinomas, two esophageal carcinomas, and two lung carcinomas. Six cases were found simultaneously with the primary malignancies. In the remaining six patients, the mean interval between MTTG and the primary malignant tumor was 6.1 years (range, 9 months to 10 years). Five patients underwent thyroidectomy. Three patients were treated with palliative treatment, including chemotherapy and radiotherapy. The clinical characteristics of the patients were listed in **Table 1**. The US features of tumors were listed in **Table 2**. Two cases showed diffuse involvement of the thyroid, and the other ten patients had focal nodules in the thyroid. The two cases with diffuse involvement showed heterogenous isoechoic or hypoechoic patterns combined with hypoechoic lines and low peripheral blood flow signal.

**Table 1:** Clinical data of patients with metastases to the thyroid gland.

No	Sex	Age (years)	Primary Tumor site	Intervals Between procedures (years)	Diagnostic Methods	US-FNA Results	Pathological type	Symptoms	Treatment
1	M	56	Esophagus	*	US-FNA	Metastasis	–	NE, cough	Chemotherapy
2	F	62	Breast	*	US-FNA, Surgery	PTC	ADC	NE	Total thyroidectomy, Chemotherapy
3	F	50	Esophagus	*	US-FNA	Metastasis	–	NE, cough	Nil
4	F	51	Lung	3	US-FNA, US-CNB	Malignant, undetermined significance	ADC	–	Chemotherapy
5	F	69	Kidney	7	Surgery	–	CCRCC	–	Total thyroidectomy
6	F	53	Breast	9	Surgery	–	ADC	–	Total thyroidectomy, Chemotherapy
7	M	66	Kidney	10	US-FNA, US-CNB	Malignant, undetermined significance	CCRCC	NE	–
8	M	48	Larynx	*	US-FNA, Surgery	Metastasis	SCC	Hoarseness	Total thyroidectomy
9	F	68	Breast	*	US-FNA, US-CNB	Metastasis	ADC	–	Nil
10	M	69	Larynx	0.75	US-FNA, Surgery	Metastasis	SCC	–	Total thyroidectomy
11	F	60	Lung	*	US-FNA	Metastasis	–	–	Nil
12	F	68	Breast	7	US-FNA	Metastasis	–	–	Chemotherapy

F, female; M, male; US, ultrasound; FNA, fine needle aspiration; CNB, core-needle biopsy; PTC, papillary thyroid carcinoma; SCC, squamous cell carcinoma; CCRCC, clear cell renal cell carcinoma; ADC, adenocarcinoma; NE: neck enlargement. *, the metastatic thyroid neoplasm was found synchronously with the primary carcinoma.

**Table 2:** Ultrasonographic characteristics of patients with metastases to the thyroid gland.

No	Forms	Locations	Max Size	Composition	Echoic Pattern	Margins	Shape	Calcification	Vascularization	Suspicious Lymph Nodes	Tumor Thrombus
1	N	Rt	46	Solid	Hypoechoic	Irregular	Parallel	–	Peripheral, high	+	–
2	N	Rt	38	Solid	Hypoechoic	Irregular	Parallel	Micro and macro	Intra-nodular, high	+	+
3	D	/	/	Solid	Heterogenous	/	/	–	Peripheral, low	–	–
4	N	Rt	32	Solid	Hypoechoic	Irregular	Parallel	Micro	Peripheral, low	+	–
5	N	Lt	58	Mixed	Hypoechoic	Circumscribed	Parallel	–	Intra-nodular, high	–	–
6	N	Lt	6.7	Solid	Hypoechoic	Irregular	N-P	–	None	–	–
7	N	Lt	62	Solid	Hypoechoic	Irregular	Parallel	–	Intra-nodular, high	+	+
8	N	Lt*	23	Solid	Hypoechoic	Irregular	Parallel	Micro	Intra-nodular, middle	+	–
9	D	/	/	Solid	Heterogenous	/	/	–	Peripheral, low	+	–
10	N	Lt	15	Solid	Hypoechoic	Irregular	Parallel		Peripheral, low	–	–
11	N	Rt	14	Solid	Hypoechoic	Irregular	Parallel		None	–	–
12	N	Lt	33	Solid	Hypoechoic	Irregular	Parallel	Micro	Intra-nodular, high	+	–

N, nodule pattern; D, diffuse pattern; Rt, right; Lt, left; Calcification, intra-tumoral calcification; Lt*, the patient had bilateral thyroid metastases and the largest nodule was in the left lobe. N-P, non-parallel; Micro, microcalcification; Macro, macrocalcification.

**Figure 1:**
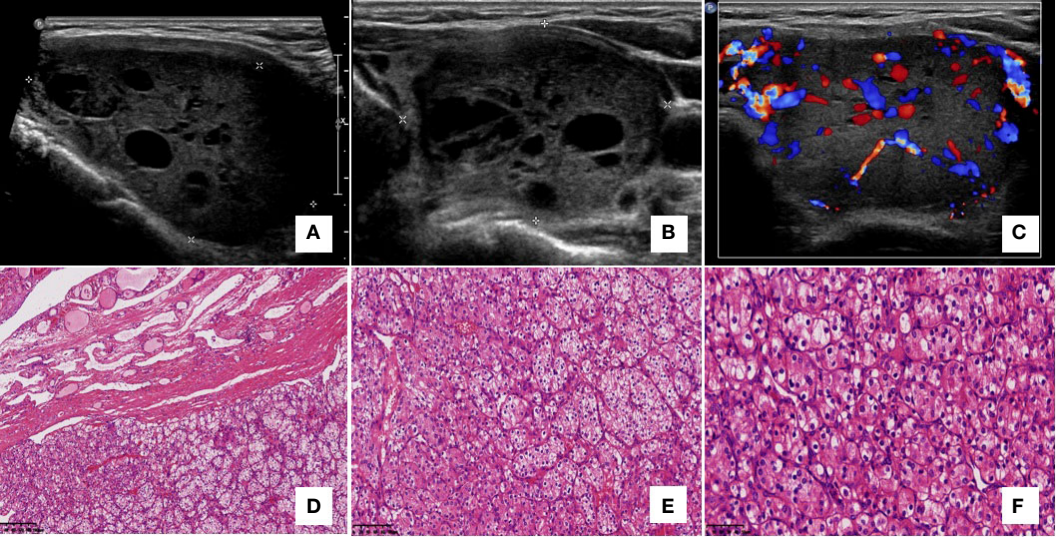
Case 5, a 69-year-old woman had a 7-year history of clear cell renal cell carcinoma (RCC). A regular and circumscribed nodule with solid and cystic composition was found on ultrasonography in the left thyroid lobe. The nodule showed a parallel orientation and no calcifications **(A, B)** with rich intra-tumoral vascularity **(C)**. No suspicious cervical lymph nodes were detected on ultrasonography. The postoperative pathology showed metastatic RCC. Histology showed a well-demarcated expanding tumor with a fibrous capsule (**D** – 100x), which had the characteristics of RCC with delicate arborizing vessels and background colloid and typical vessel formation (**E** – 200x), large vacuolated clear cytoplasm, eosinophilic cytoplasm and mild nuclear atypia (**F**– 400x), **(A–C)** ultrasonography; **(D–F)** hematoxylin-eosin staining.

**Figure 2:**
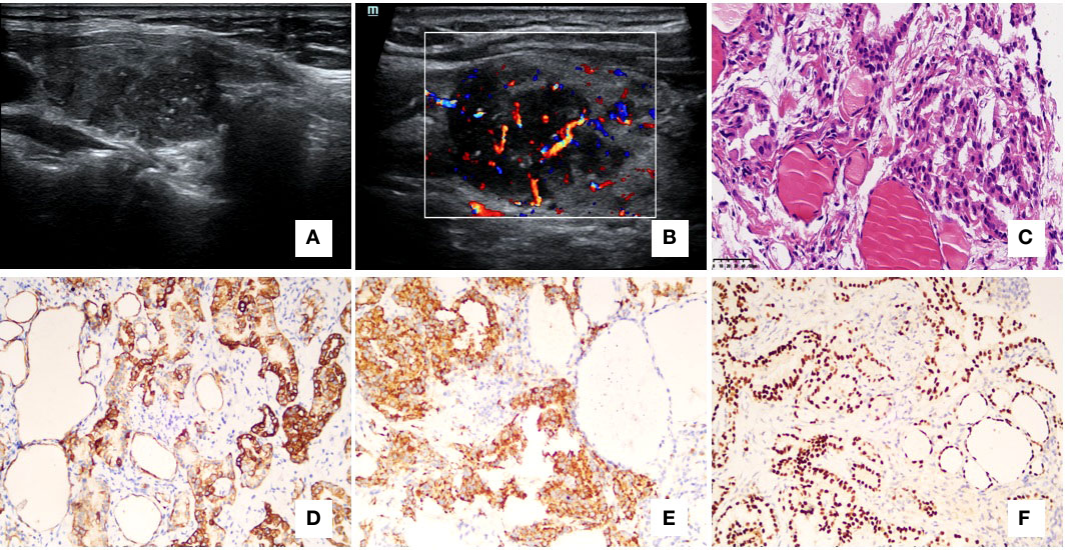
Case 4, a 51-year-old woman had a 3-year history of lung adenocarcinoma. Ultrasonography of the right lobe **(A)** showed an irregular, hypoechoic nodule with microcalcifications. Doppler ultrasonography showed rich intra-tumoral vascularity **(B)**. The cytology result was atypia with an undetermined source, and then a core needle biopsy was performed. The histology showed the papillary and tubular-acinar architecture and malignant cuboidal to columnar tumor cells with mild nuclear atypia [**(C)** – 400x]. In immunohistochemistry, the malignant cells were positive for CK7 (**D** – 200x), Napsin A [**(E)** – 200x], and TTF1 (**F** – 200x). **(A, B)** ultrasonography; **(C)** hematoxylin-eosin staining; **(D–F)** immunohistochemistry.

**Figure 3:**
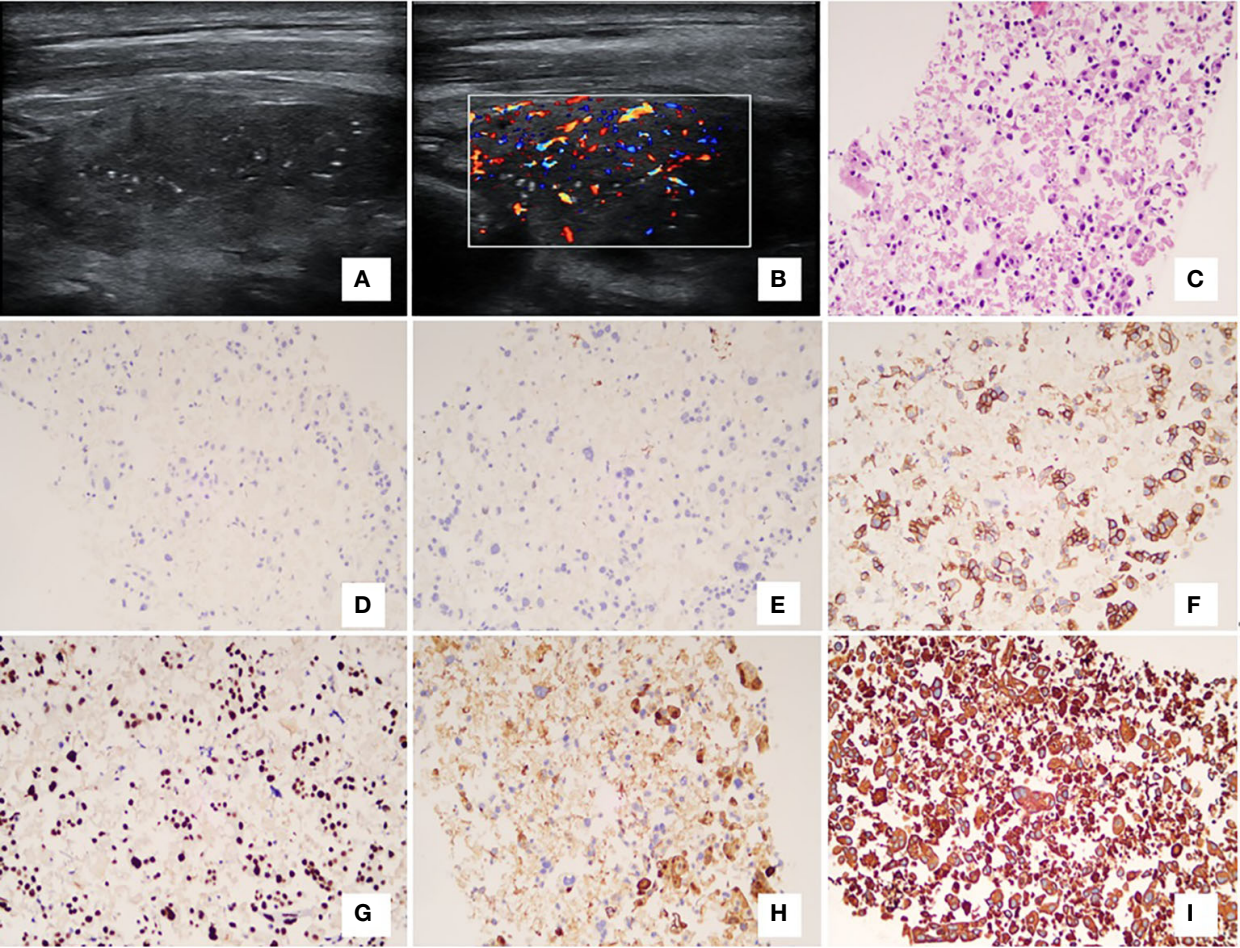
Case12, a 68-year-old female patient had a 7-year history of malignant breast tumor. Ultrasonography showed an irregular and hypoechoic nodule with micro-calcifications **(A)**. On color Doppler, it showed intra-nodular high vascularization **(B)**. The patient underwent fine-needle aspiration cytology. The cell mass exhibited scattered malignant tumor cells with necrosis, large hyperchromatic nuclei, and irregular nuclear membranes [**(C)** – 200x]. In immunohistochemistry, the malignant cells showed negative expressions of estrogen receptor [**(D)** – 200x] and progesterone receptor [**(E)** – 200x], and positive for HER2 (**F** – 200x), GATA-3 [**(G)** – 200x], GALACTIN-3 (**H** – 200x), and AE1/AE3 (**(I)**– 200x). **(A, B)** ultrasonography; **(C)** hematoxylin-eosin staining; **(D–I)** immunohistochemistry.

The mean maximum diameter of the ten nodules was 31.6 mm (range, 7 - 58 mm). Among the ten local nodules, the maximum diameter was <1 cm in one case (10%), ≥1 cm but <3 cm in four cases (40%), and ≥3 cm in five cases (50%). The location of the metastatic nodule was in the left lobe of the thyroid (n=5) and the right lobe (n=4). One patient (case 8) had bilateral suspicious lesions, and the largest nodule was in the left lobe. The majority of patients (9/10) had a hypoechoic irregular nodule. A mixed solid and cystic nodule with a circumscribed margin was observed in one case (case 5) metastasized from RCC (**Figure 1**), and the remaining lesions were solid. Microcalcifications were observed in three lesions (**Figures 2**, **3**), and one nodule was presented with micro- and macrocalcifications. Five nodules showed an intra-nodular blood flow, and three nodules showed peripheral blood signals on color Doppler. There were three nodules with low vascularization, five with high vascularization, and two with no blood signal. Suspicious lymph nodes were detected in six patients. Tumor thrombus in the jugular vein was documented in two patients.

FNAC was performed in 10 cases. According to the cytology results, 7 cases were diagnosed as metastases, of which four patients were diagnosed with the help of immunocytochemical analyses. Two cases were diagnosed as malignancy with unknown origin (case 4 and case 7). However, immunocytochemical analyses were not performed. One patient was diagnosed with papillary thyroid carcinoma with partly anaplastic carcinoma (case 2). Total thyroidectomy was performed in five patients.

## Discussion

In the present study, the male-to-female ratio was 1:2, and 41.7% of our patients presented with symptoms. The most frequent primary site was the breast. Half of the patients were metachronous during diagnosis of metastatic neoplasms and primary carcinoma. Three patterns of US features were found in our study. The FNAC diagnosis was correct in seven of 10 patients.

Despite the rich blood supply of the thyroid, the low incidence of MTTG is quite surprising (18). It may be attributed to the fast blood flow throughout the thyroid gland and the high concentration of oxygen and iodine, inhibiting the growth of malignant cells (19). The primary malignancies usually spread by direct invasion of adjacent organs, as well as through the bloodstream to distant organs (5, 9, 20). The male-to-female ratio in our study was 1:2, revealing a slight predominance of females for MTTG. It was similar to the result of a previous study, which reported a female-to-male ratio of about 1.4:1 (7). In our present study, one case had hoarseness, and four patients (33.3%) had neck enlargement, including two cases with cough symptoms. Papi et al. (1) reported that 72% of patients have clinical symptoms due to detectable enlarged nodules, which was much higher compared with our research. The discrepancy might be because the lesions discovered by high-resolution frequency US are smaller (5).

In most autopsy series, lung and breast are the most frequent primary sites for MTTG (6, 21). However, in clinical findings, RCC is the most frequent source (2, 5–7, 12, 22, 23). Our study showed that the most frequent primary site was breast, followed by lung, kidney, esophagus, and larynx, which was inconsistent with previous studies. One reason for the discrepancy with previous studies might be the difference in cancer prevalence among study populations (1, 11). A study based on the Asian population showed that the breast was the most common primary site of metastatic thyroid tumor (16).

In our study, metachronous metastases were observed in 6 patients. The mean interval between the initial cancer diagnosis and MTTG in the remaining patients was 6.2 years, which was similar to a previous study (7). The longest interval was ten years for the patient with RCC in our study. The reason for the long interval might be that RCC was less invasive. Therefore, metastases to the gland had more chances to be found through long-term follow-up (21), peculiarly in cases with a low clinical grade (24). Other studies also reported long time intervals in patients with primary RCC (5, 9), with the longest interval being 24 years (25). In patients with non-primary renal malignant tumors, MTTG is simultaneously diagnosed with a primary malignancy in a higher proportion compared with metastasis from malignant renal tumors (4). Therefore, we suggested that newly detected nodules in patients with a history of RCC should be of concern, even if the primary RCC was resected many years ago.

Two types of US appearances of MTTG have been reported, including diffuse and mass-forming patterns (8, 11, 12, 15). Two cases presented diffuse patterns in our study. The diffuse pattern has been rarely reported in previous studies (8, 10, 15, 26). The primary sites of the diffuse pattern were various, including lung, breast, colon, stomach, biliary duct, and penile (8, 15). In this study, the primary sites were the breast and esophagus. The US features were similar to those in Kim’s study (8). Kim et al. (8) reported 13 cases of diffuse metastases to the thyroid gland, eight of which were involved bilateral lobes, which presented as a diffuse goiter with heterogeneously hypoechoic or isoechoic echogenicity with internal hypoechoic lines. In our study, diffuse enlargement in two cases occurred in bilateral lobes. Zhou et al. have reported that diffuse calcifications in diffuse heterogeneous tissue were observed in six of eight patients with MTTG from primary breast malignancies (26). However, it was not observed in other studies (8, 10). Most patients with diffuse patterns presented with cervical lymphadenopathy (8, 10), which was similar to the findings in our study. These results indicate that diffuse patterns in the thyroid gland with enlarged lymph nodes can be a clue for metastases to the thyroid when there is a history of the malignant tumor.

The mass-forming pattern is generally presented as a focal hypoechoic nodule with an irregular margin, sometimes accompanied by enlarged lymph nodes (1, 8, 11, 14, 23), which is consistent with our study. These characteristics are similar to those of primary malignant thyroid nodules (27). However, microcalcifications are not commonly detected in mass-forming types (1, 14, 28). The nodules with suspicious US features without microcalcifications may be suggestive of MTTG in patients with a history of non-thyroid malignancies (11, 14). Nevertheless, our studies demonstrated three cases with microcalcifications and one case with micro-and macrocalcifications, accounting for 40% of mass-forming types, which was inconsistent with previous studies. Therefore, we suggested that microcalcification might not be a reliable indicator to differentiate primary tumors from MTTG. Yoon et al. (11) found that peripheral blood flow was observed in 85.7% of mass-forming MTTG, and Saito et al. (15) reported that MTTG usually showed a high blood supply. According to our results, the vascularization intensity and characteristics of MTTG were nonspecific. No blood signal was also found in two lesions.

There were two cases of clear cell RCC metastasized to the thyroid in our study, one of which was oval in shape with a circumscribed margin and a mixture of cystic and solid composition, similar to a benign nodule. Hypoechoic nodule with a circumscribed character was also found in some studies (14, 29). Song (29) found that all the secondary thyroid neoplasms from RCC were hypoechoic and oval-shaped, of which 88.9% of nodules showed circumscribed margins. In macroscopic findings, these nodules were well circumscribed and encapsulated (24). The mixed composition may reflect histologic findings of hemorrhages, necrosis (30), or liquefaction (31). Based on the benign sonographic findings of the case in our study, it was difficult to distinguish from primary benign thyroid nodules. A history of malignancy may be helpful in the diagnosis. However, the final diagnosis still needs pathological examination.

Traditionally, CNB can give an accurate result without further examinations (32). In our present study, CNB was performed in four patients, and these results were consistent with cytological results. As a less invasive method, FNAC is also a valuable tool for diagnosing MTTG (21). The difference between malignant cells’ ‘foreign’ population and the normal follicular epithelium in the thyroid is essential for diagnosing MTTG (2).

However, there is a diagnostic dilemma for MTTG from other primary malignancies since the cytology features of high-grade malignancy of metastasis are similar to those of anaplastic thyroid carcinoma (2, 6). Moreover, the extravasated erythrocytes of a hypervascular lesion increase the difficulty of the diagnosis (33). In case 7 and case 4, which were metastatic from clear cell RCC and lung carcinoma, FNAC results showed malignancy with undetermined sources. The false-negative outcomes of FNAC in metastatic RCC were also obtained in previous studies (7, 12, 29), and the most common type is clear cell RCC (34). The microscopy shows clear cytoplasm consisting of lipidic and glycogenic composition (31) and distinct cell membrane with pleomorphic nuclei (23), and these cytological characteristics are similar to those of Hürthle cells or even macrophages (17). Similarly, the metastatic breast tumor may be confused with thyroid carcinoma in an appropriate cellular content with tiny abnormality in nuclear (23). In case 2, the thyroid nodule was diagnosed as papillary thyroid carcinoma with partly anaplastic carcinoma. Therefore, cytology morphology alone may sometimes be insufficient to make an accurate diagnosis (1). The immunohistochemical analysis is helpful for differential diagnosis (12).

With the development of immunohistochemical stains in recent years, these auxiliary techniques can help diagnose metastasis when the microscopy is not inclusive (7, 20). The appropriate antibodies for the known history of carcinoma can be selected for immunohistochemistry (2, 7). In this study, four patients underwent immunohistochemical stains with FNAC samples, all of which had accurate results. This might help patients with widespread metastases avoid unnecessary surgical resections. However, the radiologist should be aware of collecting enough materials for the immunohistochemical when performing FNAC.

Despite its uncommon clinical presentation in surgical practice (21), the incidence of the autopsy of MTTG can be up to 24% in patients who died of non-thyroid malignancies (4, 35). However, MTTG was discovered in 0.16% of patients who underwent FNAC and 1.9% of all thyroid FNAC categorized as malignant in a multicenter study from Europe and the USA (17). As a safe, accurate, minimally invasive, and cost-effective diagnostic approach (36), FNAC with cell block analyzed by immunohistochemical stains is more acceptable and may lead to the increased frequency of MTTG.

For patients with widely spread metastases and aggressive primary disease, FNAC of MTTG can avoid unnecessary surgeries due to poor prognoses. However, surgeries may get control of the central neck pressure symptoms and even long-term cure in an isolated metastasis during the follow-up of indolent primary malignancies (5, 21, 37). Prudent scales of the metastatic tumors’ load, the biology of primary malignancies, and comorbidities should be made for personalized decisions (21). FNAC can achieve an earlier and more accurate diagnosis for the multidisciplinary team to communicate subsequent management.

There are several limitations of our study. First, with the wide use of FNAC, a molecular approach (20) can be combined with cytology and immunocytochemistry to help diagnose, but we didn’t use it in our present study. Second, cytological results were the final diagnostic method in four of 10 patients, all of whom had widespread metastases, and further aggressive methods, such as core-needle biopsy or thyroidectomy, could not be undergone. Besides, the sample size was small, and a longer follow-up should be considered to evaluate treatment outcome and survival.

## Conclusions

Most of the metastatic thyroid cancer has similar ultrasound features to primary thyroid cancer. In some cases with atypical US features, ultrasound diagnosis should be combined with the medical history. US-guided FNAC with cell block may provide an accurate diagnosis of metastatic thyroid cancer, leading to personalized approaches from the multidisciplinary team.

## Data availability statement

The original contributions presented in the study are included in the article/supplementary material. Further inquiries can be directed to the corresponding authors.

## Ethics statement

The studies involving human participants were reviewed and approved by Institutional Review Board of Shanghai Ruijin Hospital. The ethics committee waived the requirement of written informed consent for participation.

## Author contributions

ZT was the major contributor in writing the manuscript, conducting and designing the study. JZ, XW and LG participated in the image annotation, evaluation, and study design. The two corresponding authors provided clinical information on all cases. All authors contributed to the article and approved the submitted version.

## Conflict of interest

The authors declare that the research was conducted in the absence of any commercial or financial relationships that could be construed as a potential conflict of interest.

## Publisher’s note

All claims expressed in this article are solely those of the authors and do not necessarily represent those of their affiliated organizations, or those of the publisher, the editors and the reviewers. Any product that may be evaluated in this article, or claim that may be made by its manufacturer, is not guaranteed or endorsed by the publisher.
